# Prevalence and risk factors of occupational neck pain in Chinese male fighter pilots: a cross-sectional study based on questionnaire and cervical sagittal alignment

**DOI:** 10.3389/fpubh.2023.1226930

**Published:** 2023-10-31

**Authors:** Fengyuan Yang, Zhong Wang, Hongxing Zhang, Bowen Xie, Hui Zhao, Lu Gan, Tengfei Li, Jing Zhang, Zhiqiang Chen, Tianqi Li, Xiaogang Huang, Yufei Chen, Junjie Du

**Affiliations:** ^1^Department of Orthopedics, Air Force Medical Center of the PLA, Beijing, China; ^2^Graduate School of Medicine, China Medical University, Shenyang, China; ^3^Department of Spine Surgery, Central Hospital of Dalian University of Technology, Dalian, China; ^4^Division of Spine Surgery, Department of Orthopedics, Daping Hospital of Army Medical University, Chongqing, China; ^5^Air Force Clinical College, The Fifth School of Clinical Medicine, Anhui Medical University, Hefei, China; ^6^Institute for Traffic Medicine, Daping Hospital, Army Medical University, Chongqing, China

**Keywords:** neck pain, neck injury, sagittal balance, spinal curvatures, military pilots, risk factors, injury prevention, injury assessment

## Abstract

**Background:**

Neck pain (NP) is a common musculoskeletal disorder among fighter pilots and has become a rising concern due to its detrimental impact on military combat effectiveness. The occurrence of NP is influenced by a variety of factors, but less attention has been paid to the association of NP with demographic, occupational, and cervical sagittal characteristics in this group. This study aimed to investigate the prevalence and risk factors of NP in Chinese male fighter pilots using a questionnaire and cervical sagittal measurements.

**Methods:**

Demographic and flight-related data, as well as musculoskeletal pain information, were gathered from Chinese male fighter pilots via a self-report questionnaire. Cervical sagittal parameters were measured and subtypes were classified using standardized lateral cervical radiographs. Differences in various factors between the case and control groups were analyzed using *t*-tests or chi-square tests. Binary logistic regressions were conducted to explore potential risk factors contributing to NP. Predictors were presented as crude odds ratios (CORs) and adjusted odds ratios (AORs), along with their respective 95% confidence intervals (CIs).

**Results:**

A total of 185 male fighter pilots were included in this cross-sectional study. Among them, 96 (51.9%) reported experiencing NP within the previous 12 months. The multivariate regression analysis revealed that continuous flight training (AOR: 4.695, 95% CI: 2.226–9.901, *p* < 0.001), shoulder pain (AOR: 11.891, 95% CI: 4.671–30.268, *p* < 0.001), and low back pain (AOR: 3.452, 95% CI: 1.600–7.446, *p* = 0.002) were significantly associated with NP.

**Conclusion:**

The high 12-month prevalence of NP among Chinese male fighter pilots confirms the existence of this growing problem. Continuous flight training, shoulder pain, and low back pain have significant negative effects on pilots’ neck health. Effective strategies are necessary to establish appropriate training schedules to reduce NP, and a more holistic perspective on musculoskeletal protection is needed. Given that spinal integrated balance and compensatory mechanisms may maintain individuals in a subclinical state, predicting the incidence of NP in fighter pilots based solely on sagittal characteristics in the cervical region may be inadequate.

## 1. Introduction

Non-combat injuries are the leading cause of pilot attrition and military discharge in modern warfare (1). Spine-related pain, such as neck pain (NP) and its association with occupational hazards, is a well-documented complaint among military pilots (2). Unlike helicopter or transport aircraft pilots, fighter pilots usually experience high G-forces, repetitive head and neck flexion and rotation, and added weight from the helmet and oxygen equipment (3–5), which increase the load on the cervical vertebrae, especially during neck rotation and extension (6, 7). These specific occupational factors place fighter pilots at a higher risk of spinal injury presenting as NP than helicopter and transport pilots (8), not to mention the general population (9).

The prevalence of NP in fighter pilots has increased significantly due to the ever-increasing intensity of flight training (10, 11), with reports of up to 83% in a 12-month period and up to 97% over the course of a career (3). While some cases of ligamentous disruption, vertebral fractures, and disk pathologies that require surgery have been documented (12, 13), most fighter pilots self-report mild to moderate non-specific NP (14, 15). However, such NP is often reported to have a negative impact on a pilot’s physical and mental health (16), manifesting as impaired attention and concentration, poor motor control, postural instability, inability to perform in-flight maneuvers, task interruption, and temporary or permanent grounding (13, 17). These harmful impacts on individual health and operational capability can lead to substantial losses in military interests (12), especially through attrition and early career termination (17). It is noteworthy to consider that training an operational military pilot costs around $9 million (12), and even higher at $15.2 million for a single fighter pilot (17). Therefore, effective and efficient preventive measures are needed to reduce the high incidence of NP among military pilots. Combined with appropriate medical management, this may enable military pilots to avoid suffering long-term pain and disability, thus ensuring good military strength. However, before developing and recommending preventive strategies and keeping medical readiness, injury assessment models must first identify prevalence rates and etiological factors (17, 18).

Cervical sagittal alignment and balance play a crucial role in maintaining physiological function of the cervical spine and serve as critical indicators in evaluating cervical degeneration. These include sagittal curvature, sagittal displacement, and various cervicothoracic junction parameters (19–22). Previous research has shown differences in cervical sagittal parameters, specifically cervical lordosis (C2-C7 angle) (15), T1 slope (19, 23, 24), and C2-7 sagittal vertical axis (SVA) (19, 24), between healthy individuals and patients with NP. Additionally, pain is prevalent in other regions of the body, such as the shoulder and lower back (25), which may worsen the impact of NP. In addition, age, inappropriate BMI, and smoking habit may be associated with a higher risk of developing NP (26, 27). These predictive factors may resemble those found in previous research on NP in the general population, but no study has comprehensively investigated the factors associated with the occurrence of NP in the population of fighter pilots with regard to the above areas. At present, research on occupational factors has primarily focused on flying time or experience, such as total, annual, or weekly flying hours (4, 15, 17, 28–33) and duration of occupational exposures (31, 34, 35). However, there has been neglect in investigating the impact of flight training schedules on NP, such as continuous or non-continuous flight training. The purpose of this cross-sectional study was to assess the prevalence of occupational NP in Chinese fighter pilots and to determine associated factors by analyzing demographic and occupational information and cervical sagittal characteristics.

## 2. Methods

### 2.1. Study design and participants

This cross-sectional study was designed to investigate the prevalence and associated risks of NP in Chinese male fighter pilots using a questionnaire survey and radiological measurements. The Ethics Committee of the Air Force Medical Center of the People’s Liberation Army of China (PLA) approved the study (No. 2023-11-PJ01) and it was conducted in accordance with the Helsinki Declaration. Before the study commenced, written informed consent was obtained from all participants. The participants were recruited at the Air Force Medical Center using independently controlled quota sampling based on military theater command distribution, and data collection began from August 2021 to November 2022. The survey was anonymous and self-administered, with a paper copy of the questionnaire distributed to each enrolled participant. The X-ray examinations were performed by the Radiology Department of the Air Force Medical Center of the PLA.

All male participants were actively serving in Air Force military units in the Five Theater Commands of the PLA. They were certified fighter pilots aged between 20 and 48. The exclusion criteria for the participants were as follows: (1) any current or past history of known trauma or surgery to the spine and joints, signs of neurological deficit, or structural lesions; (2) under medical treatment for physical pain; (3) systemic disease affecting the musculoskeletal system (e.g., osteoarthritis, rheumatoid arthritis, etc.); and (4) absence from flying for more than four consecutive weeks in the previous 12 months (e.g., vacation, study, etc.). A formula for estimating prevalence study was employed to determine the sample size for this study. An expected NP prevalence of 51% (10) and a margin of error of 15% were considered, resulting in a required sample size of 172. To account for potential power loss due to invalid responses or radiographs, an additional 15% was added, bringing the final number of invited pilots to 198. A total of 198 male pilots participated in the study, with 41 serving in the Eastern Theater Command, 40 in the Southern Theater Command, 39 in the Western Theater Command, 41 in the Northern Theater Command, and 37 in the Central Theater Command. However, only 185 participants were ultimately enrolled, with 37 in the Eastern Theater Command, 38 in the Southern Theater Command, 36 in the Western Theater Command, 38 in the Northern Theater Command, and 36 in the Central Theater Command. The dropout data were: (1) six pilots completed invalid questionnaires due to missing items; (2) seven pilots took non-standard radiographs with questionable anatomical locations or unclear image markings.

### 2.2. Questionnaire measures

The complete list of items in the questionnaire can be found in Appendix. The study’s questionnaire comprised three sections as follows:

Section 1: Baseline characteristics of the participants such as age, height, weight, and smoking status. Weight of the participants was measured to the nearest 0.1 kg using a digital scale while wearing light clothing. Standing height was measured to the nearest 0.5 cm. Body mass index (BMI) was calculated as weight in kilograms divided by height in meters squared, to the nearest 0.1 kg/m^2^. Current smokers were defined as having smoked at least 100 cigarettes in their lifetime and having smoked in the past 30 days, with two response options (yes/no).
Section 2: This section was based on records of aircraft piloting. The occupational data included total flying hours (flying hours in a career) and annual flying hours (flying hours in the past 12 months). As total or annual flying time may only provide an ambiguous description of cumulative chronic exposure within a career or 12 months, we defined an indicator reflecting flight training schedules: continuous flight training refers to ≥6 h per week (1) for more than 4 consecutive weeks in the past 12 months, with two response options (yes/no). Service units were surveyed to confirm the practicability of quota sampling according to military theater distribution. However, this data were not used as a variable in the study due to limited access to display military details.
Section 3: This section utilized a modified version of the validated Nordic Musculoskeletal Questionnaire (1, 36) to evaluate the prevalence of musculoskeletal symptoms (pain). The body parts, including neck, shoulders, upper back, elbows, lower back, wrists/hands, hips/thighs, knees, and ankles/feet, were defined by shaded areas on body maps (Appendix). Three questions were developed for each body part, including: (1) “In your career, have you had any pain, discomfort, or numbness in this area?” (yes/no); (2) “In the past 12 months, have you had any pain, discomfort, or numbness in this area?” (yes/no); and (3) “In the past 12 months, have you been prevented from doing normal activities (e.g., work, housework, hobbies) because of this condition?” (yes/no).

A preliminary questionnaire was utilized in a prior investigation (1). To ensure that the questions were relevant and comprehensible, a board of specialists in clinical medicine, epidemiology, and aeromedicine content validated the questionnaire. Before the formal study, this pre-questionnaire was piloted with 20 people to test the language and logical order of each question, and to slightly modify the question with unclear meaning and specify the completion requirements as the final form. A question about alcohol abuse was removed because it was deemed unsuitable and unreliable for this profession. In order to obtain more statistically valid, homogeneous, and generalizable results, pilots were asked to complete baseline data (Section 1) that would be aligned with their electronic medical records at the time of enrollment (10). Additionally, to enhance reliability of flying experience and to minimize recall bias, participants were asked to report data on occupational characteristics (Section 2) according to their flight logs (10). Flight logs were completed by the pilot based on mission status, reviewed by the unit commander and flight surgeon, and provided to the medical provider at the time of the medical evaluation. In this study, NP was defined as any reported pain, discomfort, or numbness in the past 12 months that interfered with work, housework, or hobbies (37). The participants were informed of their rights and assured their privacy would be protected to minimize reporting bias. The data collection was conducted in closed rooms to ensure privacy and limit outside influences. Trained investigators were assigned to explain the questionnaire at the distribution site. The 12-month prevalence of NP was calculated as the percentage of all participants who answered “yes” to both questions (1–3) about the neck in Section 3. For statistical analysis, pilots were categorized into NP group (reporting any NP in the previous year) and non-NP group (not reporting any NP in the previous year) according to the NP presentation identified by the questions.

### 2.3. Radiographic measures

All radiographs were taken under identical conditions using the same procedure as described below. The participants stood upright and gazed straight ahead while keeping their shoulders fully relaxed and their arms naturally hanging at their sides. The cervical spine films were taken at a source-subject distance (SSD) of 150 cm with the beam centered at C4, approximately at the level of the mandibular angle (38). All subjects were positioned and imaged by the same researcher, an 8-year veteran radiologic technologist. The Luminos dRF Max (Siemens Healthcare GmbH, Erlangen, Germany) was used as the radiographic machine. The radiographs were recorded on an Imaging Clinical Information System (ICIS; version 2014.1.SU6.5, AGFA HealthCare N.V., Mortsel, Belgium) at a resolution of 1,928 × 2,308 pixels.

**Figure 1:**
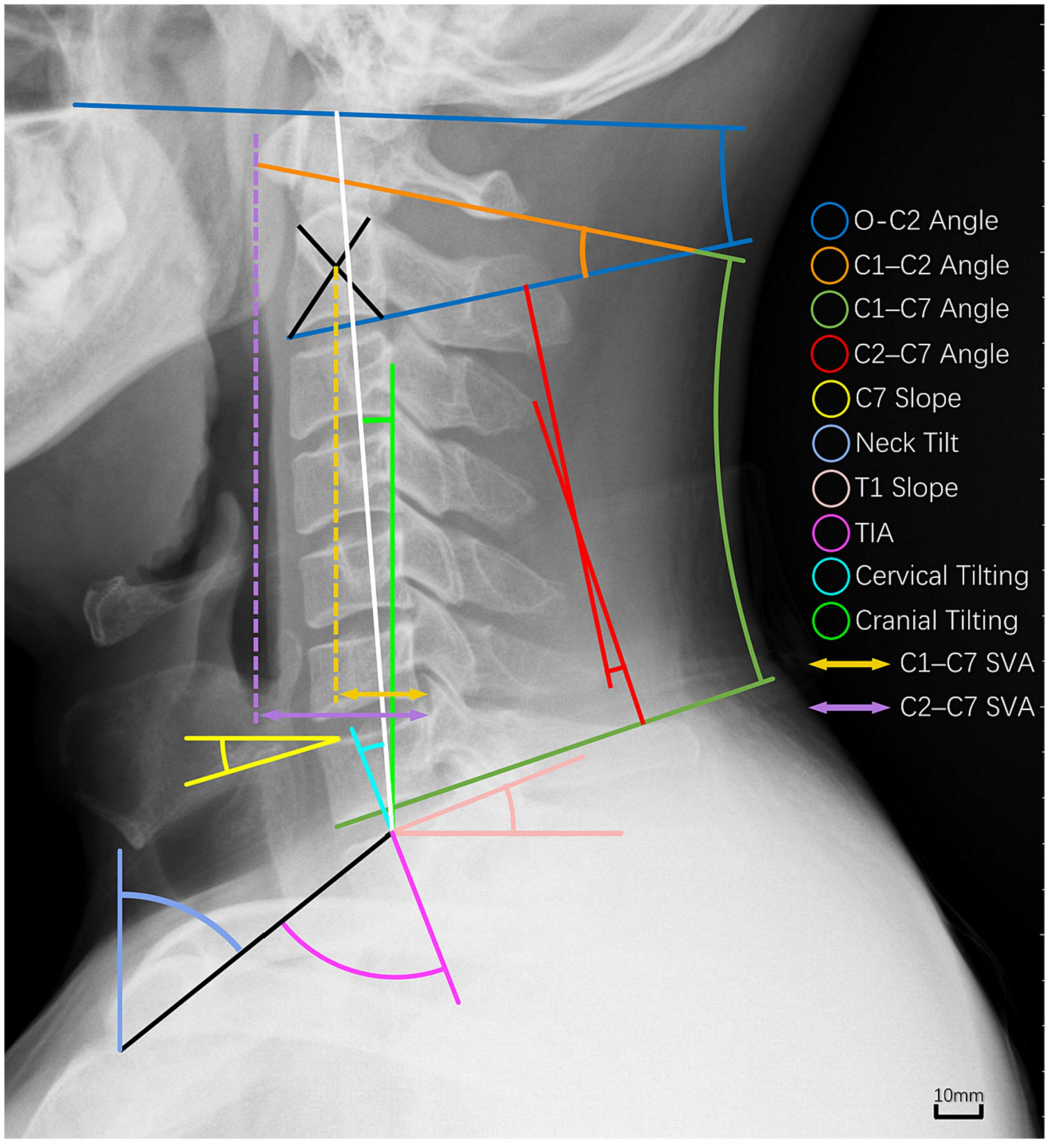
Measured cervical sagittal parameters of the study in the lateral cervical radiograph.

Cervical sagittal parameters were calculated for each participant, as displayed in Figure 1. The definition of each measured parameter (39–45) was listed in Table 1. According to the description outlined above, three experienced spine surgeons, blinded to subject grouping, independently measured all the sagittal parameters of 185 radiographs using Surgimap software (version 2.3.2.1, Nemaris, New York, NY, United States) (43). The results of these measurements were averaged to present the final data for this study. The measurements were also subject to evaluation of inter-rater reliabilities using intraclass correlation coefficients (ICCs) and average measures. The standard interpretations of the ICCs were as follows: 0.00–0.50 (poor reliability), 0.50–0.75 (moderate reliability), 0.75–0.90 (good reliability), and > 0.90 (excellent reliability) (46). Excellent reliability was observed for the parameters measured with O-C2 angle (ICC = 0.992, *p* < 0.001), C1-C2 angle (ICC = 0.980, *p* < 0.001), C1-C7 angle (ICC = 0.984, *p* < 0.001), C1-C7 SVA (ICC = 0.997, *p* < 0.001), C2-C7 angle (ICC = 0.995, *p* < 0.001), C2-C7 SVA (ICC = 0.996, *p* < 0.001), neck tilt (ICC = 0.958, *p* < 0.001), TIA (ICC = 0.958, *p* < 0.001), T1 slope (ICC = 0.973, *p* < 0.001), cervical tilting (ICC = 0.983, *p* < 0.001), cranial tilting (ICC = 0.942, *p* < 0.001), and T1S-CL (ICC = 0.983, *p* < 0.001). Good reliability was reported for the C7 slope (ICC = 0.889, *p* < 0.001).

**Table 1:** Definition of cervical sagittal parameters which were used in this study.

Parameter	Definition
O-C2 angle	The angle between McGregor’s line (the line connecting posterior edge of the hard palate to the opisthion) and the lower endplate of C2.
C1–C2 angle	The angle between the line connecting the inferior anterior arch and the inferior posterior arch of C1 and the inferior endplate of C2.
C1–C7 angle	The angle between the line connecting the inferior anterior arch and the inferior posterior arch of C1 and the inferior endplate of C7.
C1–C7 SVA	The distance from the posterosuperior corner of C7 and the perpendicular to the center of the anterior edge of the C1 body.
C2-7 angle	The angle between the C2 lower endplate and the C7 lower endplate. The C2-7 angle is also referred to as C2-7 lordosis (CL).
C2-7 SVA	The distance from the posterosuperior corner of C7 and the perpendicular to the center of the C2 body.
C7 slope	The angle between a horizontal line and the upper endplate of C7.
T1 slope (T1S)	The angle between a horizontal line and the superior endplate of T1 on a standing lateral radiograph (T1S = cervical tilting + cranial tilting).
Neck tilt (NT)	The angle between the line connecting the center of the T1 upper endplate and the top of the sternum and the vertical line extending from the sternum tip.
Thoracic inlet angle (TIA)	The angle between the line connecting the center of the T1 superior endplate and the top of the sternum and the vertical line extending from the center of the T1 superior endplate (TIA = T1S + NT).
Cervical tilting	The angle between the line through and perpendicular to the center of the T1 upper plate and the line from the center of the T1 upper plate to the tip of the dens.
Cranial tilting	The angle between the line from the center of the T1 upper endplate to the dens and the perpendicular through the center of the T1 upper endplate.
T1S-CL	The T1S minus the C2-7 lordosis (CL).

O-C2, Occiput-C2; SVA, Sagittal vertical axis; and CL, C2-7 lordosis.

**Figure 2:**
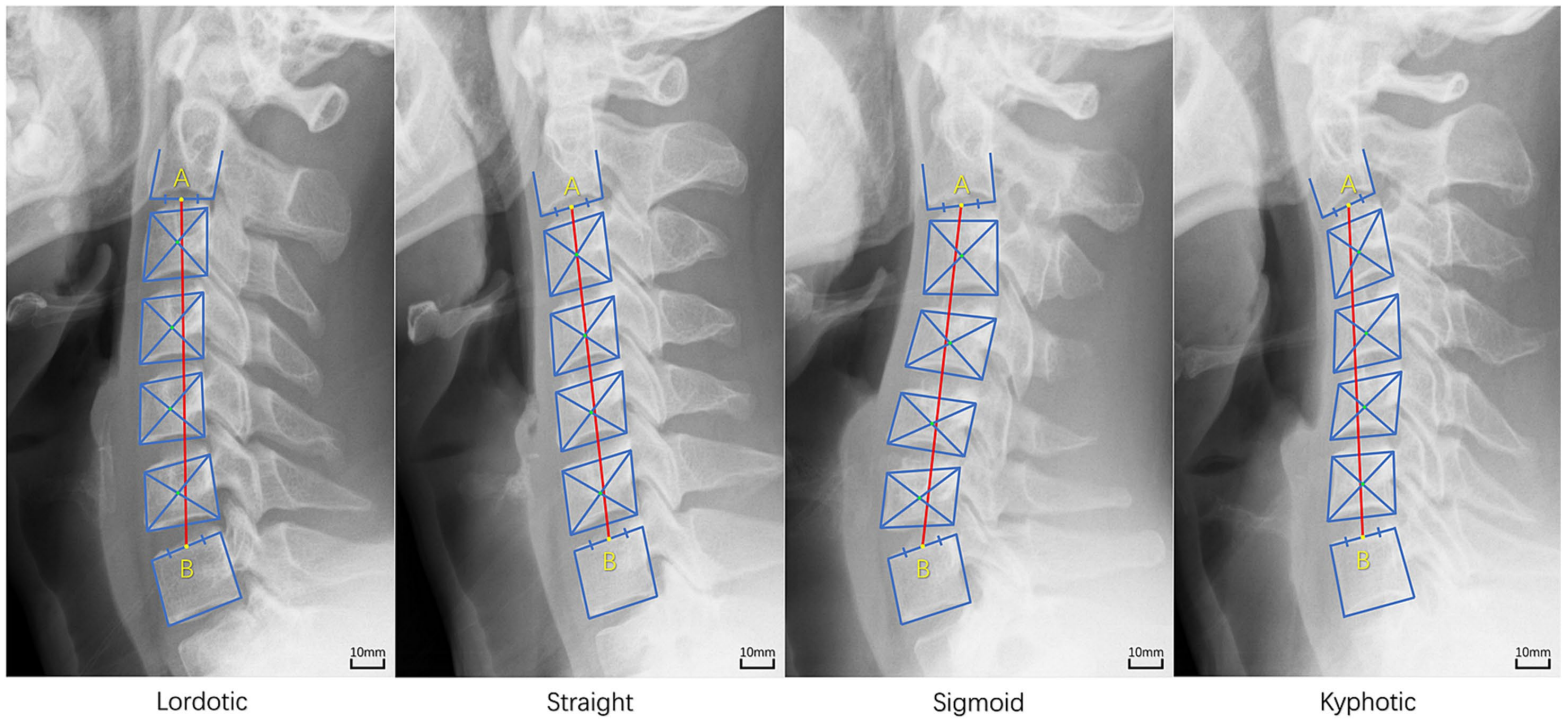
The method of subtype classification of cervical sagittal alignment [modified method of Toyama et al. (47)]. Lordotic: all centroids (green colored dot) are anterior to the line AB (red colored line) and the distance between at least one centroid and the line AB is 2 mm or more; Straight: the distance between the line AB and each centroid is less than 2 mm; Sigmoid: some centroids are anterior and some are posterior to the line AB and the distance between the line AB and at least one centroid is 2 mm or more; Kyphotic: all centroids are posterior to the line AB and the distance between at least one centroid and the line AB is 2 mm or more.

Cervical sagittal alignment classifications were evaluated using a modified method of Toyama et al. (47). The contour tangents to the four sides of the C3-C6 vertebral bodies were constructed by connecting adjacent corners with a straight line. Each pair of diagonally opposite corners where adjacent contour tangents intersected was connected by a line, respectively, (Figure 2). The intersection of these two lines is the vertebral centroid. Line AB was constructed to connect midpoint A on the inferior surface of C2 and midpoint B on the superior surface of C7. The alignment was then classified as lordotic, straight, sigmoid, or kyphotic based on the relative positions of the centroids to the line AB (Figure 2). The cervical sagittal alignment of the 185 radiographs was independently classified into lordotic, straight, sigmoid, or kyphotic groups by the same three orthopedic surgeons using Surgimap software as described above. The final subtype for each participant was determined by majority rule. Inter-rater agreement among the classifications was evaluated using the Fleiss kappa coefficient. The kappa values were categorized as follows: 0.00–0.20 (slight agreement), 0.21–0.40 (fair agreement), 0.41–0.60 (moderate agreement), 0.61–0.80 (substantial agreement), and 0.81–1.00 (almost perfect agreement) (48). In this study, the inter-rater agreement for the classifications demonstrated almost perfect agreement with a Fleiss kappa coefficient of 0.889 (*p* < 0.001).

### 2.4. Statistical analysis

Statistical analyses were conducted using SPSS software (version 26.0, Chicago, United States). The questionnaire and radiographic measurement results were presented as mean ± standard deviation for quantitative data and as absolute values with percentages for qualitative data. Data normality was assessed using the Kolmogorov–Smirnov test (1). Differences in normally distributed quantitative data between the NP and non-NP groups were assessed using independent two-sample *t*-tests. Cohen’s *d* values were calculated using G*Power software to evaluate significant effects (49). The Cohen’s d values were categorized as follows: 0.00–0.10 (negligible effect), 0.10–0.20 (small effect), 0.20–0.50 (medium effect), 0.50–0.80 (large effect), and > 0.80 (very large effect) (50). Differences in non-normally distributed quantitative or qualitative data between the NP and non-NP groups were assessed using chi-square tests with two-tailed Cramer’s V coefficient. The Cramer’s V values were categorized as follows: 0.00–0.10 (small association), 0.10–0.30 (medium associations), 0.30–0.50 (large association), and > 0.50 (very large association) (51). For multiple comparisons of NP prevalence among cervical sagittal subtypes, chi-square tests were conducted followed by Bonferroni analyses (50). Univariate and multivariate logistic regression analyses were performed to identify variables influencing the prevalence of NP in fighter pilots. All possible variables were included in the multivariate logistic regression, and a stepwise elimination procedure was applied to control for potential confounders to determine the simplest and most accurate regression model. Crude odds ratios (CORs) and adjusted odds ratios (AORs) along with their corresponding 95% confidence intervals (CIs) were reported. The model fit was assessed using Hosmer-Lemeshow goodness of fit test. The Hosmer-Lemeshow statistic indicates a poor fit if the significance value is less than 0.05 (50). All *p* values were two-tailed, and *p* < 0.05 was considered statistically significant.

## 3. Results

### 3.1. Demographic and occupational characteristics of the participants and pain prevalence

Table 2 shows the background data of the study participants. A total of 185 male fighter pilots were included in this study, with a mean age of 28.5 ± 6.0 years, mean height of 173.8 ± 3.6 cm, mean body weight of 70.8 ± 7.1 kg, mean BMI of 23.4 ± 2.1 kg/m^2^, mean total flying time of 996.5 ± 835.6 h, and mean annual flying time of 151.9 ± 34.3 h. Almost half of the pilots reported continuous flight training in the past year (57.3%), while the other half did not (42.7%). Current smoking was reported by 70 (37.8%) pilots, and 116 (62.7%) had a BMI of less than 24 kg/m^2^.

**Table 2:** Background data for all participants and for NP and non-NP groups.

	Total (*N* = 185)	NP group (*N* = 96)	Non-NP group (*N* = 89)	Cohen’s d or Cramer’s V	*p* value
Demographic data					
Age (year)	28.5 ± 6.0	28.8 ± 5.6	28.1 ± 6.4	0.124	0.400^a^
Height (cm)	173.8 ± 3.6	173.9 ± 3.8	173.7 ± 3.4	0.043	0.772^a^
Weight (kg)	70.8 ± 7.1	71.3 ± 7.4	70.3 ± 6.7	0.137	0.352^a^
BMI (kg/m^2^)	23.4 ± 2.1	23.5 ± 2.1	23.3 ± 2.0	0.127	0.389^a^
< 24 kg/m^2^ (*n*)	116 (62.7%)	59 (50.9%)	57 (49.1%)		
≥ 24 kg/m^2^ (*n*)	69 (37.3%)	37 (53.6%)	32 (46.4%)		
Current smoking					
Yes (*n*)	70 (37.8%)	42 (60.0%)	28 (40.0%)	0.127	0.085^b^
No (*n*)	115 (62.2%)	54 (47.0%)	61 (53.0%)		
Occupational data					
Total flying hours (h)	996.5 ± 835.6	1041.4 ± 821.5	948.1 ± 852.6	0.112	0.449^a^
Annual flying hours (h)	151.9 ± 34.3	156.3 ± 35.5	147.2 ± 32.6	0.269	0.069^a^
< 150 h (*n*)	88 (47.6%)	44 (50.0%)	44 (50.0%)		
≥ 150 h (*n*)	97 (52.4%)	52 (53.6%)	45 (46.4%)		
Continuous flight training					
Yes (*n*)	106 (57.3%)	73 (68.9%)	33 (31.1%)	0.394	<0.001^b^
No (*n*)	79 (42.7%)	23 (29.1%)	56 (70.9%)		
Pain in other body areas					
Shoulder pain					
Yes (*n*)	59 (31.9%)	52 (88.1%)	7 (11.9%)	0.496	<0.001^b^
No (*n*)	126 (68.1%)	44 (34.9%)	82 (65.1%)		
Low back pain					
Yes (*n*)	72 (38.9)	48 (66.7)	24 (33.3)	0.236	0.001^b^
No (*n*)	113 (61.1)	48 (42.5)	65 (57.5)		

^a^Independent two-sample *t*-test was used; ^b^Chi-square test was used.

Among 185 participants, 96 (51.9%) reported NP (95% CI: 44.6–59.2%), 72 (38.9%) reported low back pain (95% CI: 31.8–46.0%), and 59 (31.9%) reported shoulder pain (95% CI: 25.1–38.7%) in the past 12 months (Table 2).

As demonstrated in Table 2, the incidence of NP was significantly higher in pilots with continuous flight training than in pilots without continuous flight training (68.9 vs. 29.1%, *p* < 0.001, Cramer’s V = 0.394, *p* < 0.001); however, the NP and non-NP groups did not differ significantly in total flying hours (*p* = 0.449) and annual flying hours (*p* = 0.069). In addition, significant differences were found in the incidence of NP according to pain in other areas of the body. The incidence of NP was 88.1% in the patients with shoulder pain (*p* < 0.001, Cramer’s V = 0.496, *p* < 0.001) and 66.7% in the patients with low back pain (*p* = 0.001, Cramer’s V = 0.236, *p* < 0.001). There were no significant differences in age, height, weight, BMI, or smoking history between the NP and non-NP groups.

### 3.2. Cervical sagittal characteristics of the participants

The cervical sagittal characteristics of the subjects, including parameters and alignment subtypes, are shown in Table 3. The distribution of cervical sagittal alignment subtypes in the total cohort was as follows: lordotic subtype in 61 (33.0%), straight subtype in 80 (43.2%), sigmoid subtype in 20 (10.9%), and kyphotic subtype in 24 (13.0%). In other words, the lordotic subtype accounted for 33.0% of the total cohort compared to 67.0% for the non-lordotic subtype.

**Table 3:** Cervical sagittal characteristics for all participants and for NP and non-NP groups.

	Total (*N* = 185)	NP group (*N* = 96)	Non-NP group (*N* = 89)	Cohen’s *d* or Cramer’s V	*p* value
Cervical sagittal parameter					
O-C2 angle (deg)	15.1 ± 6.6	15.6 ± 6.4	14.5 ± 6.7	0.163	0.269^a^
C1-C2 angle (deg)	24.5 ± 5.8	24.8 ± 5.9	24.1 ± 5.7	0.133	0.369^a^
C1-C7 angle (deg)	28.8 ± 9.1	29.0 ± 9.9	28.5 ± 8.3	0.061	0.681^a^
C1-C7 SVA (mm)	33.7 ± 10.7	33.4 ± 11.1	34.1 ± 10.4	0.069	0.641^a^
C2-C7 angle (deg)	5.1 ± 9.5	5.1 ± 9.8	5.1 ± 9.2	0.002	0.987^a^
C2-C7 SVA (mm)	18.6 ± 8.6	18.3 ± 8.9	18.9 ± 8.4	0.070	0.636^a^
C7 slope (deg)	17.5 ± 6.6	17.2 ± 5.2	17.8 ± 7.8	0.087	0.554^a^
Neck tilt (deg)	46.3 ± 6.1	46.1 ± 5.4	46.5 ± 6.8	0.059	0.692^a^
TIA (deg)	67.8 ± 7.5	67.7 ± 6.7	68.0 ± 8.4	0.041	0.780^a^
T1 slope (deg)	21.5 ± 5.4	21.5 ± 5.2	21.5 ± 5.2	0.009	0.953^a^
T1S-CL (deg)	16.4 ± 7.7	16.4 ± 7.8	16.3 ± 7.7	0.004	0.978^a^
Cervical tilting (deg)	18.5 ± 5.9	18.7 ± 6.3	18.3 ± 5.4	0.067	0.648^a^
Cranial tilting (deg)	3.0 ± 4.3	2.8 ± 4.3	3.3 ± 4.3	0.113	0.442^a^
Cervical sagittal subtype					
Lordotic subtype (*n*)	61 (33.0%)	29 (47.5%)	32 (52.5%)	0.074	0.801^b^
Straight subtype (*n*)	80 (43.2%)	43 (53.8%)	37 (46.2%)		
Sigmoid subtype (*n*)	20 (10.8%)	10 (50.0%)	10 (50.0%)		
Kyphotic subtype (*n*)	24 (13.0%)	14 (58.3%)	10 (41.7%)		

^a^Independent two-sample *t*-test was used; ^b^Chi-square test was used. Deg, degree; mm, millimeter.

As shown in Table 3, no significant differences were found between participants with and without NP in O-C2 angle, C1-C2 angle, C1-C7 angle, C1-C7 SVA, C2-C7 angle, C2-C7 SVA, C7 slope, neck tilt, TIA, T1 slope, T1S-CL, cervical tilting, or cranial tilting. Multiple comparisons analysis revealed that there were no significant differences in the incidences of NP according to cervical sagittal subtypes (*p* = 0.81, Cramer’s V = 0.074, *p* = 0.81). The incidence of NP was 47.5% in the lordotic group, 53.8% in the straight group, 50.0% in the sigmoid group, and 58.3% in the kyphotic group.

### 3.3. Risk factors associated with NP

Unadjusted and adjusted analyses using logistic regression were performed to assess risk factors associated with NP among the study participants (Table 4). Multivariate regression analysis revealed that continuous flight training, shoulder pain, and low back pain were significantly predictive of NP. However, the following factors were not significantly associated with the incidence of NP: age, height, weight, BMI, smoking, total flying time, annual flying time, cervical sagittal parameters, and subtypes. Participants with continuous flight training were 4.695 times more likely to have NP than those without continuous flight training (AOR: 4.695, 95% CI: 2.226–9.901, *p* < 0.001). In addition, shoulder pain (AOR: 11.891, 95% CI: 4.671–30.268, *p* < 0.001) and low back pain (AOR: 3.452, 95% CI: 1.600–7.446, *p* = 0.002) were associated with the incidence of NP.

**Table 4:** Logistic regression analysis of risk factors potentially associated with NP.

Variables	Univariate analysis		Multivariate analysis	
	COR (95% CI)	*p* value	AOR (95% CI)	*p* value
Demographic data				
Age (years)	1.021 (0.973–1.072)	0.398		
Height (cm)	1.012 (0.935–1.096)	0.770		
Weight (kg)	1.020 (0.979–1.063)	0.351		
BMI (kg/m^2^)	1.064 (0.925–1.223)	0.388		
Current smoking (Yes vs. No)	1.694 (0.928–3.095)	0.086		
Occupational data				
Total flying hours (h)	1.000 (1.000–1.000)	0.447		
Annual flying hours (h)	1.008 (0.999–1.017)	0.072		
Continuous flight training (Yes vs. No)	5.386 (2.851–10.175)	<0.001	4.695 (2.226–9.901)	<0.001
Pain in other body areas				
Shoulder pain (Yes vs. No)	13.844 (5.800–33.043)	<0.001	11.891 (4.671–30.268)	<0.001
Low back pain (Yes vs. No)	2.708 (1.463–5.014)	0.002	3.452 (1.600–7.446)	0.002
Cervical sagittal parameter				
O-C2 angle (deg)	1.025 (0.981–1.072)	0.268		
C1-C2 angle (deg)	1.023 (0.973–1.076)	0.367		
C1-C7 angle (deg)	1.007 (0.975–1.039)	0.679		
C1-C7 SVA (mm)	0.994 (0.967–1.021)	0.639		
C2-C7 angle (deg)	1.000 (0.970–1.031)	0.987		
C2-C7 SVA (mm)	0.992 (0.959–1.026)	0.634		
C7 slope (deg)	0.987 (0.943–1.032)	0.554		
Neck tilt (deg)	0.990 (0.945–1.038)	0.688		
TIA (deg)	0.994 (0.957–1.033)	0.777		
T1 slope (deg)	1.002 (0.950–1.057)	0.953		
T1S-CL (deg)	1.001 (0.964–1.039)	0.978		
Cervical tilting (deg)	1.012 (0.963–1.063)	0.646		
Cranial tilting (deg)	0.974 (0.910–1.042)	0.440		
Cervical sagittal subtype				
Straight subtype (vs. Lordotic subtype)	1.282 (0.658–2.500)	0.465		
Sigmoid subtype (vs. Lordotic subtype)	1.103 (0.402–3.031)	0.849		
Kyphotic subtype (vs. Lordotic subtype)	1.545 (0.595–4.012)	0.372		

COR, Crude odds ratio; AOR, Adjusted odds ratio; CI, Confidence interval.

## 4. Discussion

### 4.1. Main findings

The aim of this study was to determine the incidence of occupational NP among fighter pilots in the Chinese Air Force and to evaluate potential risk factors by analyzing demographic and occupational data as well as cervical sagittal measurements. The main finding of the study was that NP in pilots was positively associated with continuous flight training, shoulder pain, and low back pain, but there was no evidence of an association between measured cervical sagittal parameters and subtypes and NP. These results could provide supportive evidence for maintaining spinal health and preventing injury in military pilots.

### 4.2. Epidemiology of NP in fighter pilots

In this cross-sectional study, a high prevalence of musculoskeletal conditions was found, with 51.9% of respondents reporting prominent NP compared to a mean prevalence of 37% in the general population aged 17–70 years (9). Consistent with some studies, NP prevalence in fighter pilots ranged from 47 to 83% (3, 7, 15, 30, 52, 53). In contrast, lower prevalence of NP was reported in other studies (1, 4, 54, 55). The variations in prevalence observed across studies may be attributed to discrepancies in the target populations, time intervals considered, or criteria used to characterize pain and associated symptoms (15, 17). For example, differences in the time frames used to define NP are evident. A study by Vanderbeek et al. (56) found that the 3-month prevalence of NP (51%) was lower than the 12-month prevalence (64%) in fighter pilots. This discrepancy may be attributable to the shorter time frame used to define NP, which may have decreased recall bias. Another illustration is whether or not medical treatment was sought. Yang et al. (1) reported that NP necessitating medical care had a 3-month prevalence rate of 30%, whereas Ang et al. (57) did not take into account seeking care, which had a 3-month prevalence rate of 53%. It should be noted that certain military pilots may hesitate to report experiencing pain and seek medical assistance due to concerns over flight restrictions (10). This could potentially affect the described prevalence of self-reported pain. Consequently, the lack of standardized definitions has been identified as a limitation in these studies.

The gaps could be addressed through Delphi studies, which are likely to establish uniform definitions of both NP and neck regions to improve the accuracy of future results (10). We suggest identifying specific population characteristics, such as age, gender, aircraft type, flight experience, and any other relevant demographic or occupational variables, as a first step. Then, it is recommended to define NP using recognized diagnostic criteria or standardized measurement tools that are commonly used in evaluating NP. The Dutch Musculoskeletal Questionnaire, Neck Disability Index (NDI), or Visual Analog Scale (VAS) are examples of such tools that can effectively identify the anatomical location, severity, frequency, and duration of pain. Body maps with shaded areas or self-reported markings can be used to indicate the specific regions of discomfort. Consider implementing a severity grading system and establishing a timeframe for NP to capture variations in severity and frequency of symptoms. It is worth noting that whether the definition should include criteria for functional impairment or limitations in performing specific tasks related to flight duties should be determined. Additionally, it is necessary to evaluate the extent to which the injury or pain requires medical assistance. Thirdly, one should ensure that the defined criteria are validated and consistent with established clinical or research standards. It is also crucial that the definition is effectively communicated to researchers, healthcare providers, and other stakeholders involved in the study or management of NP in this population.

### 4.3. Do demographic characteristics affect the incidence of NP in fighter pilots?

As people age, their risk of developing NP in the general population might increase due to inadequate body mass index (BMI) and smoking habits (26, 27). However, the relationship between demographic factors and NP in fighter pilots remains inconclusive, likely due to the impact of occupational and physical functional characteristics, as well as social-psychological factors such as a high volume of flight missions, prolonged computer or desk work (28, 54), reduced neck strength or torque (7), and mental fatigue or anger (54).

Some recent studies have shown that age is a risk factor for NP in fighter pilots (4, 28). However, our study found no significant association between age and NP, which is consistent with other reports (7, 31, 54). While increasing age is a non-modifiable risk factor for NP in general occupational groups (58), numerous individual-related and flight-specific factors could be confounded by age for military pilots. For example, young pilots, such as trainees, typically fly low to medium performance aircraft, such as the K-8 and J-7. These pilots do not exhibit any pathological changes in the spine due to low cumulative load exposures. However, they have limited flight experience, lack awareness of effective pre- and in-flight precautions, such as warming up with range of motion and isometrics and placing their head against the seat, and are not sufficiently trained for G-load resistance. Pilots between the ages of 30 and 40 demonstrate optimal flight skills, possess knowledge of preventative measures for neck injuries, and maintain excellent physical fitness. However, many of these pilots operate high-performance fighter aircraft, such as the J-16 and J-20, which impose high peak loads during numerous flight missions. As a result, the cumulative loads have caused gradual degeneration of the spine. For pilots over 40 years old, flight training volume is reduced gradually. Flight skills and preventive measures remain mature, and rest and recovery time is longer. However, the physical function has deteriorated, making it difficult to withstand the burden of flight loads on the body, with degenerative spinal disease appearing gradually. Thus, in this study, the association between age and NP may be attenuated by the interaction of both positive and negative factors associated with the age factor.

Neck pain is generally not directly caused by smoking. However, smoking may indirectly contribute to certain factors such as reduced blood flow, impaired healing, degenerative disk disease, coughing and breathing problems, and lifestyle factors that may increase the risk of NP (59). Unlike age, smoking is a modifiable factor that can be reduced or stopped (58). Our study discovered a greater occurrence of smoking within the NP group compared to the non-NP group. However, this difference was not significant, and several previous studies also failed to discover a significant association between smoking and NP (17, 54). We assessed smoking through a yes/no question concerning the past 30-day and lifetime tobacco usage, a crude measure of this form of exposure. By classifying individuals who formerly smoked as non-smokers and those who smoke only a few cigarettes per month or day as smokers, the exposure characterization may be imprecise. This possible crude classification of exposure is likely non-differential, potentially weakening the associations.

Consistent with previous studies (32, 33), our study did not find a significant association between NP and BMI. While BMI is a measure of body fat based on an individual’s weight and height, it is not directly related to NP, nor does it take into account the distribution of body fat. Individuals with higher muscle mass may have a greater BMI without excess body fat. Conversely, someone with a lower BMI may still have a larger percentage of body fat concentrated in specific regions, such as the neck. Fat accumulation around the neck may be linked to NP, but BMI alone does not determine its occurrence. Future studies on NP in this population should consider incorporating additional anthropometric measures such as body fat percentage, fat distribution, and muscle mass. It is worth noting that the average BMI of pilots in our sample was below 24, which may imply lower muscle mass in younger pilots. This may be due to loosely supervised physical training programs, as well as frequent deployments and relocations that prevent pilots from having regular access to the gym for training and make it difficult to obtain counterbalanced training equipment.

The present study did not demonstrate a significant relationship between height and NP, in contrast to the findings of a previous study (28). Ergonomically, the impact of height on NP appears to be twofold, as both extremely tall and short individuals are at higher risk. Shorter individuals may need to lift their arms more extensively, while taller individuals may need to lean their head forward more often, underlining the importance of appropriate height in confined cockpits. Nevertheless, as aircraft and equipment ergonomics continue to improve, it appears that this effect is decreasing.

### 4.4. Do fighter pilots with shoulder pain and low back pain have an increased risk of NP?

In the present study, shoulder pain and low back pain were discovered to be independent risk factors for NP in fighter pilots. This outcome suggests a relationship between shoulder pain, low back pain, and NP, potentially associated with flight posture. As pilots sustain a seated position and stable lower body, there is an increased requirement for push-pull movements with the upper extremities (1). The position required to grip the handle with the hands and fingers results in static contraction of the neck and shoulder muscles, which act as stabilizers to maintain the arms at a perpendicular angle (15). Shoulder pain or muscle fatigue may cause the neck muscles, such as the upper trapezius and scalene, to assist in elevating or shrugging the shoulder to stabilize and control the scapula and arms. This compensation principle weakens the arm and shoulder and strains the neck muscles excessively, leading to pain. It appears to apply to the lumbar region as well. Muscles that act on the spine, including erector spinae and multifidus, may tire from continuous or repeated exposure to high G-forces, prolonged static postures or both. This could alter the sitting posture by increasing the kyphosis in the lumbar region and potentially changing the regular curvature in the proximal spine area (34). To maintain trunk balance and a forward gaze, the pilot may need to compensate by further extending the neck. This scenario could leave the pilot more susceptible to neck and lower back injuries and pain. Therefore, it is clear that the musculoskeletal system should be analyzed holistically. We should pay close attention to the possibility of NP in individuals who are suffering from shoulder or low back pain. Physical exercise, particularly comprehensive training that incorporates both endurance and strength, should be prioritized as it has been proven to have significant protective effects against NP (52, 60). Ergonomic adjustments to aircraft and flight equipment could potentially affect these compensatory effects, which result from interactions between distinct regional muscles. Therefore, such considerations must be incorporated into new aircraft and equipment designs.

### 4.5. Is NP related to flying time and training schedules?

On one hand, the total number of flying hours can partially indicate the duration of being in a particular occupational environment. Pilots’ neck muscles are significantly activated during flight, suggesting that the neck muscles are subjected to high loads (61). Although exposure to high G-forces may initially strengthen the neck (62), it is widely acknowledged that long-term exposure to such high load pressures contributes to acute or chronic episodes of NP (1, 8, 35, 63). If these loads persist over a period of time, the muscles may tire, thus potentially increasing the risk of neck muscle strain injury (13). Meanwhile, the total time spent in flight can serve as an indicator of flight experience, which to some extent may demonstrate the pilot’s flight skills or proficiency. The total flying time is an essential reference index for classifying Air Force ranks, ranging from flying cadets to top pilots. Pilots who have accumulated more flying hours are expected to possess greater flying experience or skill. Previous studies have shown a higher incidence of NP in pilots with lengthy total flying hours (4, 28, 29). However, our investigation concluded that there was no significant association between total flying hours and NP among fighter pilots. This outcome was similar to those of other published studies (30–33). The absence of significant association may be attributed to the interaction of positive and negative factors related to total flying hours, similar to the factor of age. Pilots with comparatively lower total flight time could also encounter NP if they consistently encounter high G-forces during their flights. Meanwhile, pilots who have accumulated significant flight time may not experience frequent and severe NP declines to the same degree if they have taken measures to skillfully avoid the adverse effects of high G-forces, or if they have spent more time in a state of relatively low load level flight.

Pilots who operate the same type of aircraft may encounter varying factors due to the complexity of military missions and changing flight schedules (1). Therefore, annual flying time is inadequate evidence as it only provides an imprecise description of cumulative chronic exposure within a rough time frame instead of flight training distribution. This is evident in the lack of a significant association between annual flying time and NP in our study. Flight-related NP usually appears acutely during or after flight training and takes several days to recover (16). Flying for more than 6 h per week for four consecutive weeks results in prolonged exposure to G-forces in a relatively short time and inadequate recovery time (1), which may increase the risk of NP in fighter pilots. Our findings support this perspective, demonstrating that fighter pilots who receive continuous flight training have a 4.695 times higher risk of developing occupational NP than pilots who do not receive such training. Following this risk assessment, efforts should be directed toward creating preventive measures. Intermittent flight training or distributing the training volume could potentially mitigate the incidence of NP. For instance, if the yearly training volume stays the same, flight instruction could be completed in 1 week, followed by a week of rehabilitative and physical conditioning training, alternating between the two. This organized scheduling, supervised management, and evaluation of the impacts of these interventions for NP warrant additional research in the future.

Overall, there may be some correlation between flight time data and NP for fighter pilots, but it is not the sole or primary determining factor. Other factors, such as G-forces, dynamic movements, cockpit ergonomics, physical conditioning, and individual variations, also play significant roles in the development of NP. Proper ergonomic design, rigorous pilot training, consistent exercise, and attentive posture within the cockpit can reduce the risk of NP in fighter pilots, irrespective of their total, annual, or weekly flying time (17). Furthermore, upcoming research studies must aim to identify more precise determinants of NP in this group, like exposure to high G-forces per unit time.

### 4.6. Can cervical sagittal characteristics predict NP in fighter pilots?

This study showed no significant differences in cervical sagittal parameters between individuals with and without NP. This finding aligns with previous research that found no link between NP and cervical spine curvature changes (23, 64–69). One possible explanation for the lack of differences may be that cervical sagittal parameters are not connected to NP. However, some studies have reported contradictory results. A cross-sectional study performed by Jouibari et al. (19) discovered that the NP group, among individuals with cervical lordosis, had a lower C2-7 SVA and T1 slope angle as compared to the healthy control group. This could be a compensatory action to bring the center of gravity of the head back to the spinal axis by reducing T1 slope and C2-7 SVA. However, this phenomenon varies significantly in patients with cervical kyphosis. Li et al. (24) found that the compensation mechanism of the posterior neck muscles facilitates the maintenance of cervical sagittal balance when accompanied with a lower T1 slope and smaller C2-7 SVA. Conversely, a larger C2-7 SVA with a higher T1 slope leads to a cervical malalignment that cannot be fully compensated and eventually causes NP. Therefore, a possible reason for not observing discrepancies is that the values of the cervical sagittal parameters might vary, depending on the type of cervical alignment and its associated compensation mechanism (70). Consequently, these dissimilarities cannot be detected in an unclassified sample. In addition, cervical sagittal parameter changes involve a complex compensatory mechanism that may be affected by both cervical spine degeneration and the alignment of the entire spine, including thoracic kyphosis, lumbar lordosis, and sacral slope (71–73). Thus, the compensatory site of sagittal balance in NP patients might primarily occur in the thoracic, lumbar, or pelvic segments rather than the cervical segment.

In our study, the modified method of Toyama et al. (47) was used to classify cervical sagittal alignment into four subtypes. It was found that approximately half of the individuals in each subtype did not experience NP or were subclinical in the previous 12 months, which is in accordance with several previous studies (74, 75). Although these studies utilized different classification methods, the findings suggest that NP symptoms were only slightly more common in non-lordotic subtypes than in lordotic subtypes, and that the type of cervical kyphosis was not a significant risk factor for NP (24). However, a study by Moon et al. (15) found a significant association between cervical alignment and NP in pilots. Pilots with cervical kyphosis had a significantly higher incidence of NP (81.8%) compared to those with lordosis (41.7%) or straight cervical spine (50%). These inconsistent findings suggest that the conventional view of the “normal” lordotic cervical spine (76) may not be universally applicable and that the natural sagittal alignment of the cervical spine may be morphologically diverse. An alternative viewpoint maintains that cervical misalignments always represent a pathological condition, and the lack of symptoms may indicate that the relationship between misalignment and symptoms has not yet had sufficient time to manifest. For instance, a kyphotic cervical spine increases the likelihood of developing NP due to the extra load on the neck muscles that support the weight of the head (77, 78). The compensatory response to increased load is based on excessive muscle contraction and increased tension in the small joints of the spine and intervertebral disks. This mechanism can further accelerate the progression of spinal degeneration, leading to a series of related clinical symptoms including NP, low back pain, and shoulder pain (79).

Spinal balance goes beyond the fixed morphological alignment of vertebrae in momentary imaging, and it should be regarded as a synergistic somatic balance of the nervous, muscular, and skeletal systems. We suggest that future studies on NP measure not only traditional static radiographic parameters but also precise dynamic indicators such as neuromuscular coordination, functional tasks, muscle fatigability, muscle size, kinematics, and kinetics. These additional measurements would enable a more thorough assessment of the predictive and interventional implications for NP. It is also important to note that pilots with misaligned cervical spines may still face a higher risk of experiencing more severe and frequent NP under high G-forces, such as during air combat maneuvers, or even cervical spine fractures during ejection, which could lead to paralysis. Thus, early identification of pilots with cervical pathology and special attention to their spinal health is essential. Furthermore, future research should explore the impact of upgraded equipment configurations on the frequency of NP. Implementation of lightweight head-mounted gear, protective anti-G ejection seats, and rocket-powered multi-propulsion systems may considerably enhance prevention and reduce neck injuries in military pilots.

### 4.7. Limitations

This study provides new insights into occupational NP among Chinese Air Force pilots and advances our comprehension of musculoskeletal disorders for aeromedical researchers. However, the study has some limitations to be considered. Firstly, our study may be affected by the imprecision of the prior statistical power analysis used to determine the optimal sample size. This is due to the limited research and data available on cervical sagittal characteristics in this military population using cross-sectional methodology. Although the participants came from different Air Force units and were distributed evenly across Chinese military theaters, the generalizability of the findings to the larger population of military pilots may be limited by the inability to identify the entire study population and sample distribution, as well as the relatively small sample size. Therefore, future studies with larger sample sizes and random sampling are needed. Secondly, the cross-sectional nature of the study does not allow for the determination of causality, as there is generally no evidence of the temporal relationship between risk factors and outcomes. Long-term longitudinal studies are necessary to evaluate the root causes of NP in military pilots. Thirdly, this study formed part of a more extensive research initiative, and the implementation of a standardized questionnaire during participant interviews may have resulted in the absence of particular sought-after information, including the frequency, severity, and length of musculoskeletal symptoms. Thus, future research ought to consider these facets along with symptoms originating from other bodily regions, such as hips, knees, and ankles. Additionally, the survey study should be enhanced in the succeeding phase of the intervention study by incorporating information pertaining to physical activity levels, both active and unhealthy lifestyle practices, mobility and flexibility factors, and medical consultation-seeking behavior. Finally, we measured cervical sagittal balance by taking a lateral radiograph of the cervical spine without obtaining global spinal sagittal measurements. Therefore, we were unable to determine the reciprocal influence of other spinal regions, such as the thoracic and lumbar spine. In future studies, we plan to include lateral radiographs of the entire spine to accurately measure the alignment of the spine and determine the relationship between NP and sagittal characteristics.

## 5. Conclusion

The 12-month prevalence of NP is high among Chinese male fighter pilots. Pilots experiencing low back pain and shoulder pain have a heightened risk of NP. The relationship between shoulder and low back pain and NP warrants further investigation as part of a holistic approach to musculoskeletal injury prevention. Continuous flight training schedules have a significant negative impact on pilots’ neck health. Optimizing training schedules to improve rest and prevent fatigue could potentially reduce NP in this occupation. It may be insufficient to predict the incidence of NP in fighter pilots based solely on sagittal characteristics of the cervical spine. Further elaboration of the integrated somatic balance and its compensatory mechanisms may enhance research into the causes of NP.

## Data availability statement

The raw data supporting the conclusions of this article will be made available by the authors, without undue reservation.

## Ethics statement

The studies involving humans were approved by the Ethics Committee of the Air Force Medical Center of the PLA (No. 2023-11-PJ01). The studies were conducted in accordance with the local legislation and institutional requirements. The participants provided their written informed consent to participate in this study.

## Author contributions

FY, ZW, HoZ, and BX: methodology, investigation, data curation, formal analysis, visualization, writing-original draft, and writing-review and editing. HuZ, LG, and TeL: methodology, supervision, validation, and writing-review and editing. JZ, ZC, TiL, and XH: investigation, formal analysis, and validation. YC and JD: conceptualization, methodology, project administration, funding acquisition, supervision, and writing-review and editing. All authors contributed to the article and approved the submitted version.
