# Supplementary material for: Prevalence and risk factors of occupational neck pain in Chinese male fighter pilots: a cross-sectional study based on questionnaire and cervical sagittal alignment
Source: Front Public Health. 2023 Oct 31;11:1226930. doi: 10.3389/fpubh.2023.1226930 (PMC10643867; doi:10.3389/fpubh.2023.1226930)
Supplement: Supplementary file 1 [file Presentation_1.PDF]

Questionnaire (English Version)

Section 1：General Information

Admission number：_________

Sex: ☐male ☐female

Date of birth: xx/xx/xxxx

Height：_________cm

Weight: _________kg

Current smoking (having smoked at least 100 cigarettes in lifetime and having smoked in the past 30 days): ☐no ☐yes

Section 2：Occupational Information

Service unit: _________

Aircraft type: ☐fighter, _______; ☐helicopter, _______; ☐transport, _______; ☐other, _______

Position category: ☐pilot ☐crewmember

Rank: _________

Flight rating: _________

Total flying hours in the career: _________h

Flying hours in the past 12 months: _________h

Have you experienced 6 hours or more per week for more than 4 consecutive weeks in the past 12 months? ☐no ☐yes

Section 3：Nordic Musculoskeletal Questionnaire

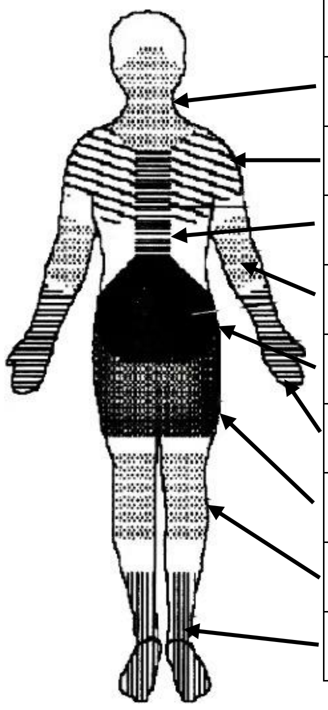

	In your career, have you had any problems (such as pain, discomfort, numbness) in:	In the past 12 months, have you had any problems (such as pain, discomfort, numbness) in:	In the past 12 months, have you been prevented from carry out normal activities (e.g., job, housework, hobbies) because of this problem in:
Neck	☐No ☐Yes	☐No ☐Yes	☐No ☐Yes
Shoulder	☐No ☐Yes	☐No ☐Yes	☐No ☐Yes
Upper Back	☐No ☐Yes	☐No ☐Yes	☐No ☐Yes
Elbow	☐No ☐Yes	☐No ☐Yes	☐No ☐Yes
Lower Back	☐No ☐Yes	☐No ☐Yes	☐No ☐Yes
Wrists/Hands	☐No ☐Yes	☐No ☐Yes	☐No ☐Yes
Hip/Thighs	☐No ☐Yes	☐No ☐Yes	☐No ☐Yes
Knees	☐No ☐Yes	☐No ☐Yes	☐No ☐Yes
Ankles/Feet	☐No ☐Yes	☐No ☐Yes	☐No ☐Yes
